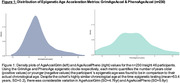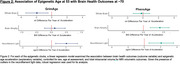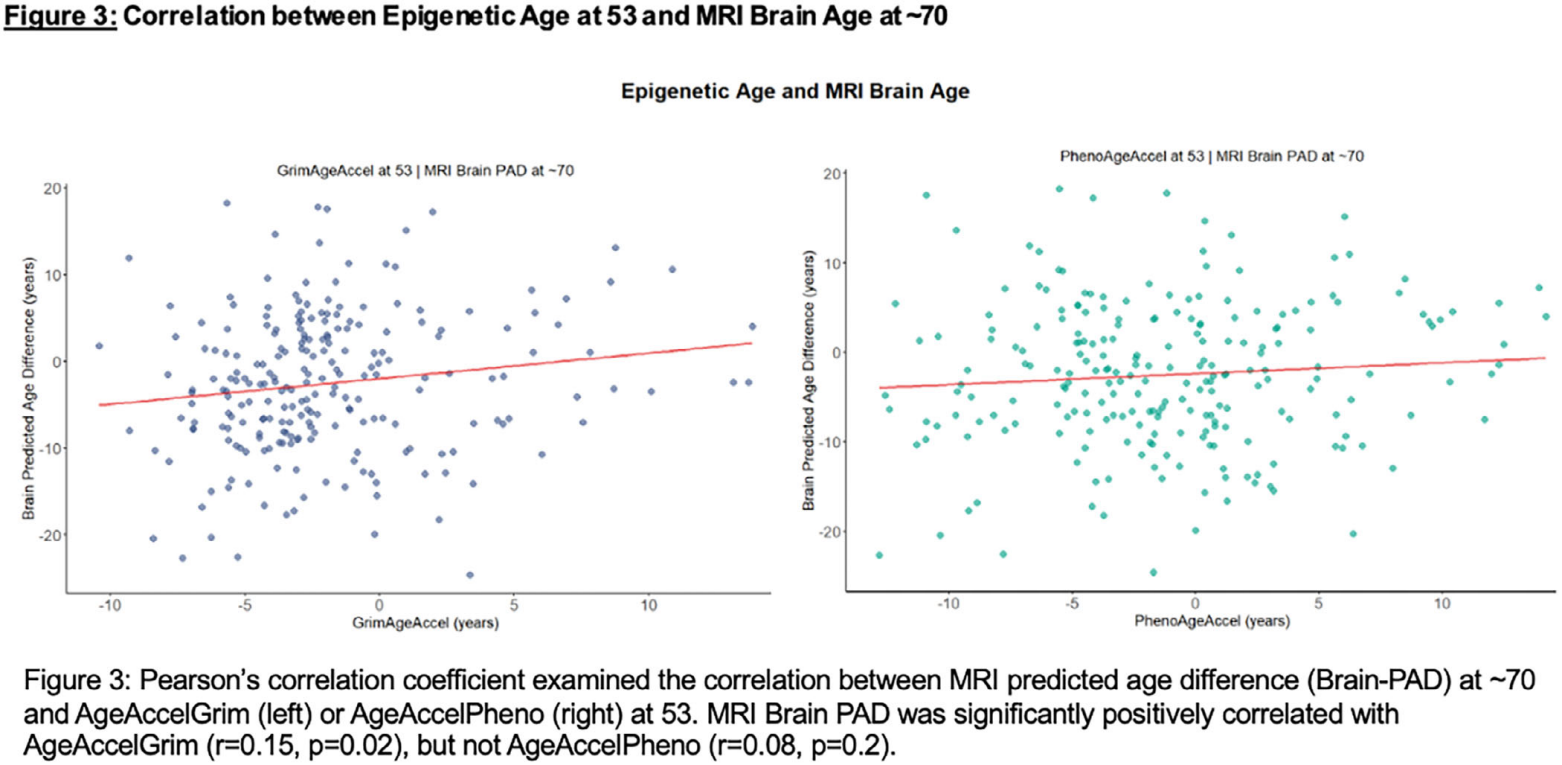# Accelerated midlife epigenetic age associates with poor later life brain health in the 1946 British Birth Cohort

**DOI:** 10.1002/alz.084231

**Published:** 2025-01-09

**Authors:** James Groves, Jane Maddock, Ashvini Keshavan, William Coath, Jennifer M Nicholas, Aaron Z Wagen, David M Cash, Frederik Barkhof, Marcus Richards, James H. Cole, Jonathan M Schott

**Affiliations:** ^1^ Dementia Research Centre, UCL Queen Square Institute of Neurology, London, London United Kingdom; ^2^ MRC Unit for Lifelong Health and Ageing at UCL, London United Kingdom; ^3^ Dementia Research Centre, UCL Queen Square Institute of Neurology, London United Kingdom; ^4^ Dementia Research Centre, UCL Queen Square Institute of Neurology, University College London, London United Kingdom; ^5^ Department of Medical Statistics, London School of Hygiene and Tropical Medicine, London United Kingdom; ^6^ Department of Clinical Movement Neurosciences, Queen Square, Institute of Neurology, London, London United Kingdom; ^7^ Neurodegeneration Biology Laboratory, The Francis Crick Institute, London, Greater London United Kingdom; ^8^ UK Dementia Research Institute at UCL, London United Kingdom; ^9^ UCL Centre for Medical Image Computing, Department of Computer Science, University College London, London United Kingdom; ^10^ MRC Unit for Lifelong Health & Ageing at UCL, London United Kingdom

## Abstract

**Background:**

Accelerated epigenetic ageing has been associated with various age‐related health outcomes, but its relevance for dementia risk prediction is unclear. We investigated whether accelerated midlife epigenetic age associates with poor later‐life brain health.

**Methods:**

Participants were 230 individuals from Insight 46, drawn from the 1946 British Birth Cohort, a population‐based study of individuals born in the first week of March 1946. DNA methylation was measured at 53 years using Infinium MethylationEPIC BeadChip and GrimAge and PhenoAge were calculated. ‘Age acceleration’ was derived for each clock (AgeAccelGrim and AgeAccelPheno), as previously. At 69‐71 years, we measured MRI whole brain and white matter hyperintensity volume, amyloid‐PET burden, plasma neurofilament light, and MRI Brain Age (with chronological age subtracted to produce the Brain‐Predicted Age Difference/Brain‐PAD). For n=176 with follow‐up MRI aged 72‐75 (n=176), whole brain atrophy was calculated using the boundary shift integral. CSF p‐tau181 was measured in n=64.

**Results:**

Despite participants’ highly similar chronological age (mean=53.4 years, SD=0.2), there was considerable variation in AgeAccelGrim (SD=4.8yr) and AgeAccelPheno (SD=5.6yr). Linear regression revealed that accelerated GrimAge was associated with smaller later‐life whole brain volume, (b=‐1.9ml, p=0.010), whilst accelerated PhenoAge was associated with greater whole brain atrophy (b=0.403, p=0.001). Neither GrimAge nor PhenoAge acceleration was associated with amyloid‐PET burden. Accelerated PhenoAge was associated with higher white matter hyperintensity burden (b=0.03, p=0.04). Acceleration in both GrimAge (b=0.4pg/ml, p=0.001) and PhenoAge (b=0.4pg/ml, p=0.001) was linked to higher plasma neurofilament light, and both AgeAccelGrim (r=0.32, p=0.010) and AgeAccelPheno (r=0.35, p=0.005) were positively correlated with CSF p‐Tau181. MRI brain age (Brain‐PAD) was significantly correlated with AgeAccelGrim (r=0.15, p=0.02) but not AgeAccelPheno (r=0.08, p=0.2).

**Conclusion:**

Acceleration of midlife epigenetic age was associated with a wide range of features of poor brain health ∼20 years later, in a population‐based sample. Epigenetic clocks may hold promise as tools for future brain health prediction.